# Genome Annotation and Catalytic Profile of *Rhodococcus rhodochrous* IEGM 107, Mono- and Diterpenoid Biotransformer

**DOI:** 10.3390/genes16070739

**Published:** 2025-06-26

**Authors:** Natalia A. Plotnitskaya, Polina Yu. Maltseva, Irina B. Ivshina

**Affiliations:** 1 Perm Federal Research Center of the Ural Branch of the Russian Academy of Sciences, 614081 Perm, Russia; inbox.98@bk.ru (P.Y.M.); ivshina@iegm.ru (I.B.I.); 2 Department of Microbiology and Immunology, Perm State University, 614068 Perm, Russia

**Keywords:** CYP450, whole genome sequencing, terpenoids, *Rhodococcus rhodochrous*

## Abstract

**Background/Objectives**: *Rhodococcus rhodochrous* IEGM 107 cells exhibit pronounced catalytic activity toward mono- and diterpenoids. However, the genetics and enzymatic foundations underlying this activity remain poorly understood. **Methods**: Using new-generation sequencing, the *R. rhodochrous* IEGM 107 whole genome was sequenced. Bioinformatic analysis and PCR were employed to identify and characterize genes, with a focus on cytochromes P450 (CYP450s). **Results**: The catalytic potential of *R rhodochrous* IEGM 107 was revealed. Its CYP450 genes were detected and analyzed, providing information on the enzymatic base of the strain related to the biotransformation of terpenoids. **Conclusions**: These findings enhance the understanding of the molecular and genetic basis for terpenoid transformations in *R. rhodochrous* actinomycetes. The results provide a foundation for future studies on gene expression and enzyme characterization aimed at developing efficient and selective biocatalysts for mono- and diterpenoid transformations.

## 1. Introduction

The biotransformation of plant terpenoids is one of the promising approaches used to obtain biologically active compounds. Actinomycetes of the genus *Rhodococcus* are highly effective catalysts for the targeted biotransformation of terpenoids of different structural groups, ranging from acyclic monoterpenoids to polycyclic triterpenoids [1]. The primary enzymes responsible for the degradation of complex organic compounds in bacterial cells are cytochromes P450 (CYP450s). However, current knowledge about the specific enzymes and corresponding genes involved in terpenoid biotransformation remains limited. To date, only a few enzymes from *Rhodococcus* species have been characterized: P450cin in *R. jostii* TMP1, which catalyzes the conversion of 1,8-cineole [2]; limonene-1,2-epoxide hydrolase (limC), involved in the transformation of limonene-1,2-epoxide and (6*S*)-carveol in *R. erythropolis* DCL14 [3,4]; and CYP108N12 and CYP108N14 from *R. globerulus*, which participate in the oxidation of *p*-cymene, (*R*)- and (*S*)-limonene, (*S*)-α-terpineol, and (*S*)-4-terpineol [5,6].

**Figure 1:**
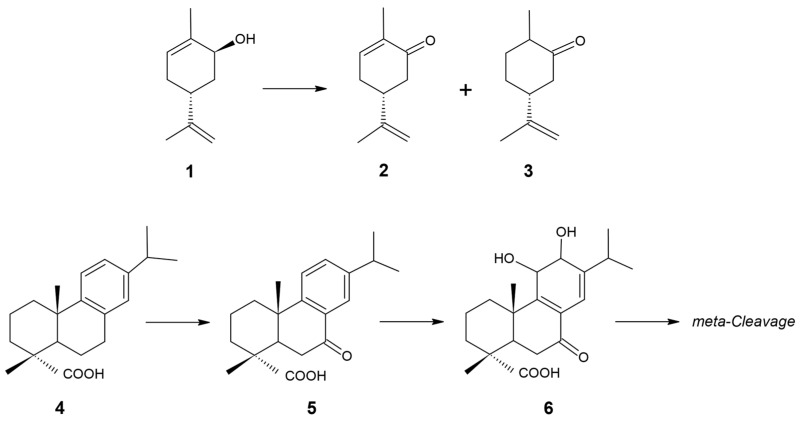
Schemes of biotransformations catalyzed by *R. rhodochrous* IEGM 107 cells: (**1**) *trans*-carveol; (**2**) carvone; (**3**) dihydrocarvone; (**4**) dehydroabietic acid; (**5**) 7-oxo-dehydroabietic acid; (**6**) 11,12-dihydroxy-7-oxo-abieta-8,13-dien-18-oic acid. Modified from [7,8].

The strain *R. rhodochrous* IEGM 107 was isolated from the water of the Dnieper River and is deposited in the Regional Specialised Collection of Alkanotrophic Microorganisms. This strain exhibits unique biotransformation activity toward both the monoterpene alcohol *trans*-carveol and the diterpenoid dehydroabietic acid (Figure 1) [7,8]. Terpenoid derivatives are recognized as highly effective bioactive compounds and serve as key precursors for pharmaceutical synthesis [9,10]. Сarvone and dihydrocarvone, for example, as components of essential oils, exhibit pronounced antibacterial and fungicidal activity [11,12]. 7-Oxo-dehydroabietic acid has long been known for its fungicidal activity [13,14]. Thus, the strain’s activity toward both *trans*-carveol and dehydroabietic acid may offer advantages for green chemistry and fine organic synthesis.

The aim of this work was to assess the catalytic potential of *R. rhodochrous* IEGM 107 and to identify the candidate genes involved in terpenoid transformation using bioinformatic tools.

## 2. Materials and Methods

### 2.1. Culture

*R. rhodochrous* IEGM 107 was isolated from the Dnieper River and is deposited in the Regional Specialized Collection of Alkanotrophic Microorganisms (acronym IEGM, WFCC number 285, WDCM number 768). In addition to transforming *trans*-carveol and dehydroabietic acid, the strain can utilize *n*-hexadecane as a sole carbon source and exhibits resistance to Pb^2+^ (10.0 mM), Cr^6+^, and Cu^2+^ (5.0 mM) (http://www.iegmcol.ru/strains/rhodoc/rhodoch/r_rhod107.html, accessed on 26 May 2025).

### 2.2. Whole Genome Sequencing

DNA extraction was performed using *R. rhodochrous* IEGM 107 cells revived from a nine-year-old lyophilized culture. The cells were cultivated in LB broth at 28 °C with shaking (160 rpm) for 24 h. Genomic DNA was isolated using the MagMAX™ Microbiome Ultra Nucleic Acid Isolation Kit (Thermo Fisher Scientific, Waltham, MA, USA) on a KingFisher™ Flex automated platform (Thermo Fisher Scientific, Waltham, MA, USA), following the manufacturer’s instructions.

A paired-end sequencing library (2 × 100 nt) was prepared using the Illumina DNA Prep Kit (Illumina, San Diego, CA, USA) and sequenced on the Illumina NovaSeq 6000 platform (Illumina, San Diego, CA, USA). Library quality was assessed via fluorescence-based quantification and the 2100 Bioanalyzer (Agilent Technologies, Santa Clara, CA, USA).

Sequencing reads were demultiplexed using Illumina bcl2fastq v2.20 (https://support.illumina.com/downloads/bcl2fastq-conversion-software-v2-20.html, accessed on 5 May 2025), and adapter trimming was performed with Skewer v. 0.2.2 [15]. FASTQ file quality was evaluated using FastQC v. 0.11.5-cegat [16], and genome assembly (contig level) was conducted using SPAdes v. 3.14.1 [17].

The whole genome sequence (WGS) of the strain is available in the NCBI database under GenBank accession number JAJNCP000000000.1.

### 2.3. Bioinformatic Analysis

For taxonomic identification, the whole genome was analyzed using the Type (Strain) Genome Server (TYGS) (https://tygs.dsmz.de, accessed on 5 May 2025) [18], a freely available bioinformatic platform that incorporates updated methodologies and features for genomic comparison [19]. Taxonomic nomenclature and synonymy were verified using the List of Prokaryotic Names with Standing in Nomenclature (LPSN, https://lpsn.dsmz.de, accessed on 5 May 2025) [19]. Pairwise genome comparisons were performed using the Genome BLAST Distance Phylogeny (GBDP) approach with the ‘trimming’ algorithm and distance formula d5 (100 distance replicates per analysis) [20]. Digital DNA–DNA hybridization (dDDH) and confidence intervals were calculated using GGDC 4.0 (Genome-to-Genome Distance Calculator 4.0, https://ggdc.dsmz.de/ggdc.php#, accessed on 5 May 2025) [19,20]. The resulting intergenomic distances were used to infer a balanced minimum evolution tree with branch support via FASTME 2.1.6.1 including SPR postprocessing [21]. Phylogenomic trees were constructed using FASTME 2.1.6.1 with SPR postprocessing and branch support estimated from 100 pseudo-bootstrap replicates. The resulting tree was midpoint-rooted [22] and visualized using PhyD3 (http://phyd3.bits.vib.be, accessed on 5 May 2025) [23]. Average Nucleotide Identity (ANI) was determined using the EZBioCloud ANI calculator (https://www.ezbiocloud.net/tools/ani, accessed on 5 May 2025) [24].

The search for the candidate genes involved in mono- and diterpenoid biotransformation, along with rRNA genes, was performed using the RAST (Rapid Annotation using Subsystem Technology, https://rast.nmpdr.org/, accessed on 5 May 2025) [25]. The analysis was performed through the automated annotation of the complete genome sequence of the biotransformer strain [26,27].

Target gene sequence comparisons were performed using the BLASTN and BLASTP tools available on the NCBI website (https://blast.ncbi.nlm.nih.gov/Blast.cgi, accessed on 8 May 2025). Biosynthetic gene cluster prediction was conducted using antiSMASH (https://antismash.secondarymetabolites.org/, accessed on 8 May 2025) [28]. Amino acid sequence analysis and metabolic pathway reconstruction were carried out using the KEGG database (Kyoto Encyclopedia of Genes and Genomes, https://www.genome.jp/kegg/, accessed on 8 May 2025) and GhostKOALA [29]. The pairwise alignment and phylogenetic tree construction of CYP450 gene products were performed using Clustal Omega (https://www.ebi.ac.uk/jdispatcher/msa/clustalo, accessed on 20 May 2025) [30], with tree visualization conducted in Jalview v. 2.11.4.1 [31]. The identification of genomic islands and the determination of GC content were performed using IslandViewer 4 [32] and GC Content Calculator (https://en.vectorbuilder.com/tool/gc-content-calculator.html, accessed on 17 June 2025), respectively.

### 2.4. PCR and Electrophoresis of Amplicons

The biomass of *R. rhodochrous* IEGM 107 was obtained after 24 h of cultivation in LB broth (Diaem, Moscow, Russia), and DNA was extracted using the ExtractDNA Blood Genomic DNA Isolation Kit (Evrogen, Moscow, Russia) following the manufacturer’s instructions. The concentration and purity of the extracted DNA were assessed using a Qubit™ Fluorometer (Thermo Fisher Scientific, Waltham, MA, USA) with a QuDye dsDNA BR kit (Lumiprobe, Moscow, Russia) and a NanoPhotometer N50 (Implen, Munich, Germany), respectively.

The purified DNA was used as a template for PCR with qPCRmix-HS SYBR (Evrogen, Moscow, Russia) and specific primers [33] on a CFX Connect™ Real-Time PCR Detection System (Bio-Rad, Hercules, CA, USA). Species-specific primers targeting the 16S rRNA gene of *R. rhodochrous* were used as a positive control. The PCR cycling conditions were applied as previously described [33].

Amplicon presence and size were verified by horizontal electrophoresis on a 1.5% agarose gel in TBE buffer, stained with GelRed (Diaem, Moscow, Russia), and visualized using a Gel Doc XR+ system (Bio-Rad, Hercules, CA, USA). PCR products (5 μL) were mixed with 0.5 μL of 4X Blue Gel Loading Dye (Evrogen, Moscow, Russia) prior to loading. A DNA length marker ranging from 50 to 700 bp (Evrogen, Moscow, Russia) was included for size estimation. Electrophoresis was performed at 70 V for 40 min.

## 3. Results and Discussion

The assembled genome of *R. rhodochrous* IEGM 107 comprised 118 contigs, totaling 5.7 Mbp in length, with an N50 of 190,984 bp, a GC content of 67.8%, and an average sequencing coverage of 357×. Genome annotation identified 5555 coding sequences (CDSs) and 56 RNA genes (Table 1).

**Figure 2:**
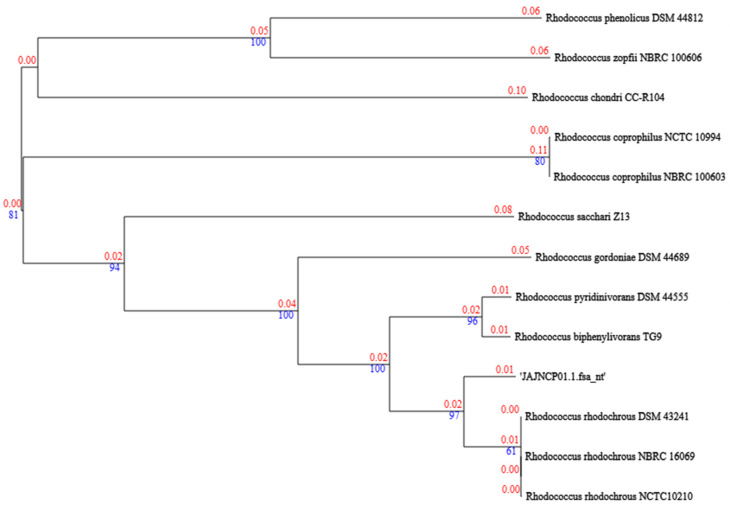
Phylogenetic tree of genomes of *R. rhodochrous* IEGM 107 (JAJNCP01.1.fsa_nt) and type strains from TYGS database. Tree inferred with FastME 2.1.6.1 [21] from GBDP distances calculated from genome sequences. Branch lengths are scaled in terms of GBDP distance formula *d_5_*. Numbers above branches are GBDP pseudo-bootstrap support values > 60% from 100 replications, with average branch support of 84.2%. Tree was rooted at midpoint [22].

Using phenotypic methods (Table S1, Supplementary Materials) [34] and an analysis of genomic characteristics (Table 2), the IEGM 107 strain was identified as belonging to the *R. rhodochrous* species (Figure 2). The resulting species and subspecies clusters are presented in Table S2 (Supplementary Materials). Eight distinct species clusters were identified, with all query strains assigned to specific clusters. Notably, the IEGM 107 strain was classified within one of the eight recognized subspecies clusters.

**Figure 3:**
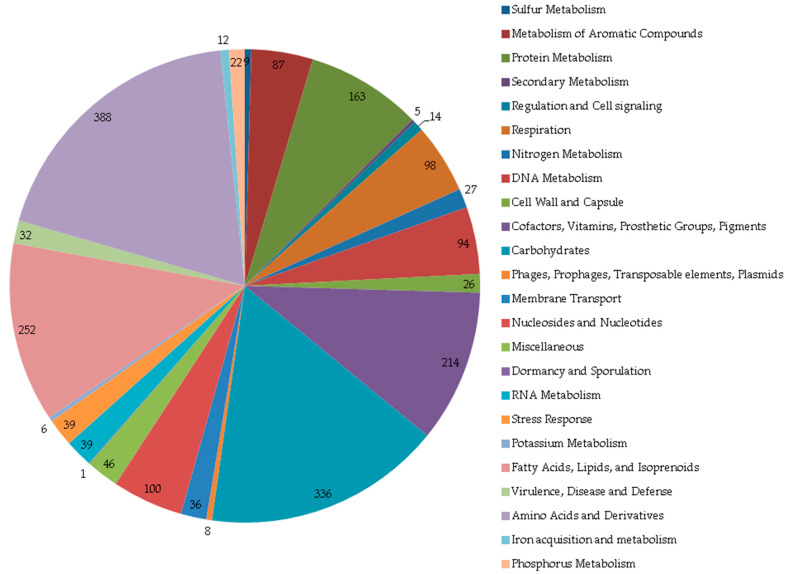
Distribution of subsystem categories in genome of *R. rhodochrous* IEGM 107. Image obtained using SEED Viewer 2.0 [35].

Among 5555 CDSs, genes of unknown and known functions were present in a 78:22 ratio. The functional category distribution of the known genes is shown in Figure 3. Notably, we identified genes encoding enzymes of the nonmevalonate pathway for isoprenoid biosynthesis, including EC 2.2.1.7, 1.1.1.267, 2.7.7.60, 2.7.1.148, 4.6.1.12, 1.17.7.1, and 1.17.1.2. Genome mining using AntiSMASH revealed biosynthetic gene clusters associated with terpene and terpene precursor biosynthesis (Table 3).

Additionally, biosynthetic gene clusters were identified for the synthesis of non-ribosomal peptide synthetases, β-lactones, ectoines, non-ribosomal peptide metallophores, non-α poly-amino acids, and type I polyketide synthases (Table 3). Within the genome of IEGM 107, 13 genes encoding non-ribosomal peptide synthetase modules for siderophore biosynthesis, 13 genes encoding polyketide synthase modules and related proteins, 1 gene for L-ectoine synthase, 4 genes for ectoine/hydroxyectoine ABC transporters, and 1 gene for ectoine hydroxylase were identified. Siderophores play a crucial role in regulating iron bioavailability, which is essential for iron-dependent enzymes including cytochrome P450 (CYP450) [36]. Polyketides and ectoines represent bioactive secondary metabolites of bacterial origin that show significant potential as therapeutic agents [37,38].

Representatives of *Rhodococcus* spp. are well known for their broad substrate specificity and ability to degrade a wide range of complex organic compounds [39]. A genomic analysis of the IEGM 107 strain identified 63 CDSs for monooxygenases, 33 for dioxygenases, 12 for hydroxylases, 7 for peroxidases, and 350 for dehydrogenases. Among the oxygenases, we detected genes encoding two alkane 1-monooxygenases, nine CYP450s, eight cyclohexanone monooxygenases, three 3-ketosteroid-9α-monooxygenases, two steroid C27-monooxygenases, two catechol 1,2-dioxygenases, three 4-hydroxyphenylpyruvate dioxygenases, two 2,3-dihydroxybiphenyl 1,2-dioxygenases, two benzoate 1,2-dioxygenases, five gentisate 1,2-dioxygenases, two protocatechuate 3,4-dioxygenases, and one lignostilbene-α,β-dioxygenase.

In addition, several genes related to sterol and steroid metabolism were identified, including six CDSs for cholesterol oxidases, one for lanosterol 14α-demethylase, four for 3-oxosteroid 1-dehydrogenases, one for 3α-hydroxysteroid dehydrogenase, one for steroid monooxygenase, two for steroid C27-monooxygenases, and three for 3-ketosteroid-9α-monooxygenases. Notably, we also identified a gene encoding limonene-1,2-epoxide hydrolase, a key enzyme in monoterpenoid metabolism. These findings highlight the extensive catalytic potential of the IEGM 107 strain.

Among the enzymes identified, CYP450s are of particular interest. CYP450 enzymes constitute a large and diverse superfamily of over 20,000 proteins that play key roles in the biosynthesis of complex natural products—including steroids, terpenes, alkaloids, flavonoids, and vitamins—as well as in drug metabolism and the degradation of environmental pollutants [40,41]. Since the 1950s, various fungal and bacterial strains harboring CYP450s have been utilized as biocatalysts in organic and medicinal chemistry applications [42].

Notably, the IEGM 107 strain catalyzes the same transformations of *trans*-carveol as *R. erythropolis* DCL14 (see Figure 1) [3,43]. While the carveol dehydrogenase from *R. erythropolis* DCL14 was identified by the authors, the complete protein sequence of carveol dehydrogenase is available in the UniProt database only for *R. jostii* RHA1 [44]. Therefore, we used this sequence for further comparative analysis. Regarding dehydroabietic acid transformations (see Figure 1), previous studies have characterized these reactions in *Pseudomonas diterpeniphila* A19-6a [45,46], with complete enzyme sequences available in UniProt. We conducted a search of these reference protein sequences in the *R. rhodochrous* IEGM 107 genome, and no high-identity matches were found. We therefore conclude that *trans*-carveol and dehydroabietic acid transformations in *R. rhodochrous* IEGM 107 are catalyzed by other functional genes and proteins.

**Figure 4:**
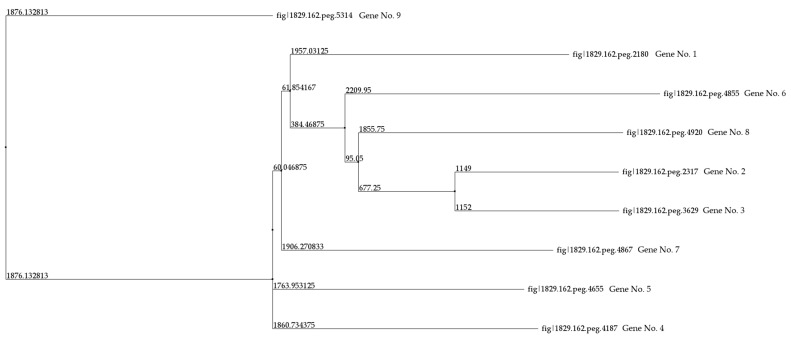
A distance tree for the pairwise alignments of the amino acid sequences of the CYP450 of *R. rhodochrous* IEGM 107. The method used to construct the tree was Neighbor Joining. Protein IDs and gene numbers are the same as those in Table 4.

In the genome of *R. rhodochrous* IEGM 107, nine genes encoding CYP450 monooxygenases and hydroxylases were identified (Table 4). A comparative analysis of nucleotide and amino acid sequences, including pairwise alignments of the latter, revealed identity levels ranging from 7.68% to 33.48%. The highest amino acid sequence identity (63.78%) was observed between CYP450s 2 and 3. Using these pairwise alignment results, a phylogenetic tree was constructed (Figure 4).

The genetic surroundings of CYP450 genes were analyzed (Table 5). In all cases, genes encoding transcriptional regulators were located either immediately upstream or in close proximity to the CYP450 genes. Furthermore, the potential for the horizontal gene transfer of the CYP450-coding genes in *R. rhodochrous* IEGM 107 was assessed. Mobile element proteins were detected adjacent to only three CYP450 genes, suggesting that horizontal gene transfer likely plays a limited role in the dissemination of CYP450 genes among *Rhodococcus* species.

To evaluate the uniqueness of the catalytic systems in *R. rhodochrous* IEGM 107, the sequences of identified genes were compared with those available in the NCBI database (Table 6). The comparison revealed that, with the exception of Genes 4 and 5, all genes are widely distributed among *R. rhodochrous* strains. Gene No. 4 is the least common, being present in only six strains, and is likely involved in complex or aromatic carbohydrate degradation. This functional inference is supported by the genomic context, which includes neighboring genes encoding 3-oxosteroid 1-dehydrogenase, isochorismatase, oxidoreductases, and a LysR family transcriptional regulator [47].

**Figure 5:**
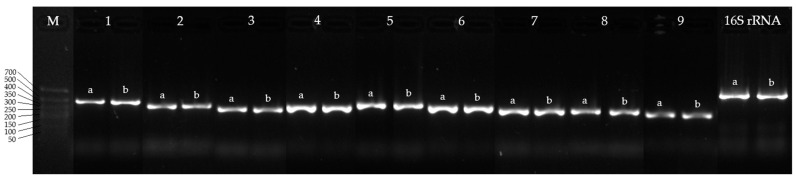
Electropherogram of PCR products of *R. rhodochrous* IEGM 107 (a) and IEGM 1362 (b) with specific primers for CYP450 genes: M, DNA length marker from 700 to 50 bp; 16S rRNA, 16S rRNA gene of *R. rhodochrous* (positive control). Gene numbers (1–9) are same as gene numbers in Table 4.

A comparative analysis of nucleotide and protein sequences (Table 6) demonstrated a high similarity between the CYP450s of IEGM 107 and those of IEGM 1362, the latter being well-characterized for its efficient biotransformation of the monoterpenoid (–)-isopulegol [48]. Given this sequence conservation, PCR was performed using primers previously designed for IEGM 1362 CYP450 genes [33]. The amplification results showed the specific detection of corresponding genes in the IEGM 107 genome, with no nonspecific products observed (Figure 5). These findings confirm that these primers can be effectively used in future studies aimed at identifying enzymes involved in terpenoid biotransformations.

Notably, CYP450 Gene 5, which shows similar nucleotide and amino acid sequences in nine and seven strains, respectively, exhibits 100% identity with CYP450 Gene 6 from the IEGM 1362 strain [33]. In the genomic context of Gene 5, genes encoding ferredoxin and ferredoxin reductase were also identified. Additionally, a mobile element protein located nearby may facilitate the dissemination of this CYP450 gene among *R. rhodochrous* strains (see Table 5). Moreover, this gene exhibits the lowest GC content (61%) among the identified CYP450 genes, compared to the 62–69% range observed for the others. Furthermore, genomic analysis revealed a genetic island containing both transposase and mobile element protein adjacent to Gene 5. These features support the potential for the horizontal transfer of this gene among *Rhodococcus* strains. These findings support evaluating the catalytic activity of the IEGM 107 strain toward (–)-isopulegol and other terpene substrates.

## 4. Conclusions

The obtained data advance our understanding of the molecular genetic basis underlying terpenoid transformation by *Rhodococcus* actinomycetes. Specifically, the identification and characterization of CYP450 enzymes and associated redox partners provide valuable insights into the enzymatic systems responsible for mono- and diterpenoid bioconversion. These findings establish a solid foundation for future investigations focused on gene expression profiling and functional validation, with the goal of identifying the key genes and enzymes involved in the highly efficient and selective biotransformation of terpenoid compounds. This research direction may ultimately contribute to the development of biocatalysts for industrial and pharmaceutical applications involving terpenoid modification.

## Figures and Tables

**Table 1:** Genome features for *R. rhodochrous* IEGM 107 assembly (according to RAST).

Feature	Value
Size, bp	5,730,104
GC content, %	67.8
N50, bp	190,984
L50	10
Number of contigs	118
Number of CDSs	5555
Number of RNAs	56
Genome coverage	357×

**Table 2:** Overall genomic characteristics of *R. rhodochrous* IEGM 107 compared with type strains from TYGS database.

Subject Type Strain	dDDH, %	G + C Content Difference, %	ANI, %
*R. rhodochrous* NBRC 16069	79.90	0.43	97.98
*R. rhodochrous* NCTC10210	79.70	0.36	97.94
*R. rhodochrous* EP4	83.50	0.11	98.21
*R. pyridinivorans* TG9	60.60	0.24	95.12
*R. pyridinivorans* DSM 44555	59.10	0.04	95.02
*R. gordoniae* NCTC 13296	41.70	0.10	90.74

**Table 3:** Biosynthetic gene clusters in the genome of *R. rhodochrous* IEGM 107.

Coding Element	Number of Clusters
Non-ribosomal peptide synthetase	10
Terpene	3
β-lactone	2
Terpene precursor	1
Ectoine	1
Non-ribosomal peptide metallophores	1
Non-α poly-amino acids like ε-polylysin	1
T1 polyketide synthase	1

**Table 4:** Genes of *R. rhodochrous* IEGM 107 encoding CYP450.

Gene No.	Protein ID	Function	Contig ID	Gene Localization	Size, bp
1	fig|1829.162.peg.2180	Cytochrome P450	JAJNCP010000006.1	99283–98072	1212
2	fig|1829.162.peg.2317	Cytochrome P450	JAJNCP010000006.1	238491–237112	1380
3	fig|1829.162.peg.3629	Cytochrome P450	JAJNCP010000012.1	67604–68950	1347
4	fig|1829.162.peg.4187	Putative cytochrome P450 hydroxylase	JAJNCP010000016.1	90221–88989	1233
5	fig|1829.162.peg.4655	Putative cytochrome P450 hydroxylase	JAJNCP010000022.1	10812–9490	1323
6	fig|1829.162.peg.4855	Cytochrome P450 monooxygenase	JAJNCP010000025.1	31024–29690	1335
7	fig|1829.162.peg.4867	Putative cytochrome P450 hydroxylase	JAJNCP010000025.1	41056–42348	1293
8	fig|1829.162.peg.4920	Putative cytochrome P450	JAJNCP010000026.1	37768–36389	1380
9	fig|1829.162.peg.5314	Putative cytochrome P450 hydroxylase	JAJNCP010000037.1	20577–18217	2361

**Table 5:** Genetic surroundings of genes encoding CYP450 enzymes in *R. rhodochrous* IEGM 107.

Gene No.	Upstream Transcriptional Regulators	Proteins Participating in Electron Transfers and Redox Reactions	Mobile Elements and Transposases	Other
1	AraC family	1,2-dihydroxycyclohexa-3,5-diene-1-carboxylate dehydrogenase; oxidoreductase FAD-binding domain protein; benzoate 1,2-dioxygenase alpha and beta subunits; 2-polyprenylphenol hydroxylase and related flavodoxin oxidoreductases; catechol 1,2-dioxygenase	No	Benzoate transport protein; benzoate MFS transporter BenK; Pca regulon regulatory protein PcaR; muconate cycloisomerase; muconolactone isomerase
2	AcrR family	4-hydroxyphenylpyruvate dioxygenase; alcohol dehydrogenase; acyl-CoA dehydrogenase; acyl-CoA dehydrogenase, putative phosphotransferase; putative short-chain dehydrogenase	No	Transcriptional regulator, AcrR family; methylated-DNA–protein-cysteine methyltransferase; RNA polymerase sigma factor RpoE; methylated-DNA–protein-cysteine methyltransferase; DNA-3-methyladenine glycosylase II; DNA polymerase I
3	IclR family	Amino acid permease; flavin monoamine oxidase-related protein; RidA/YER057c/UK114 superfamily, group 3; putative hydrolase; TTP-dependent protein, related to E1 component of pyruvate/2-oxoglutarate/acetoin dehydrogenase; 3-oxoacyl-[acyl-carrier-protein] synthase, KASIII; acyl-CoA dehydrogenase	No	Twin-arginine translocation protein TatA; two fluoride ion transporters CrcB; methyltransferase
4	LysR family	Alpha-methylacyl-CoA racemase; 3-oxosteroid 1-dehydrogenase; oxidoreductase, short-chain dehydrogenase/reductase family; enoyl-CoA hydratase; isochorismatase; oxidoreductase, short-chain dehydrogenase/reductase family	No	Conserved protein associated with acetyl-CoA C-acyltransferase
5	AcrR family	Succinate-semialdehyde dehydrogenase [NAD]; succinate-semialdehyde dehydrogenase [NADP+]; ferredoxin reductase; ferredoxin, 2Fe-2S; enoyl-CoA hydratase	Mobile element protein	Long-chain fatty acid–CoA ligase
6	Two-component transcriptional response regulator, LuxR family	Putative dioxygenase; oxidoreductase, short-chain dehydrogenase/reductase family; Gene No. 7	No	Putative esterase; metallopeptidase; possible peptidase of M23/37 family; long-chain fatty acid–CoA ligase
7	AcrR family; MarR family	Gene No. 6; epoxide hydrolase	No	Two uncharacterized MFS-type transporters
8	XRE family	Hypothetical proteins	Mobile element protein	Putative transmembrane protein; putative peptidase
9	AcrR family	Enoyl-CoA hydratase; quinone oxidoreductase; oxidoreductase, short-chain dehydrogenase/reductase family; acyl-CoA dehydrogenase	Mobile element protein	DNA-binding response regulator PhoP; two-component system phosphate sensor kinase, PhoR; L-proline/glycine betaine transporter ProP; transcriptional regulator, IclR family

**Table 6:** Homology of CYP450 genes among *R. rhodochrous* strains.

Gene No.	DNA Sequences		Amino Acid Sequences	
	Identity, %	Number of Strains	Identity, %	Number of Strains
1	81.91–100	21	65.10–100	25
2	80.30–100	20	66.75–100	51
3	79.99–100	23	63.31–100	51
4	100	6	100	6
5	79.50–100	9	66.37–100	7
6	95.43–100	18	61.56–100	33
7	95.36–100	18	97.67–100	23
8	82.74–100	10	56.36–100	26
9	75.77–100	22	59.76–100	36

A search for similar DNA and protein sequences was performed against standard NCBI databases of nucleotide collection and non-redundant protein sequences using the blastn and blastp programs, respectively. Only the percents of identity at query coverage ≥ 91% are presented. The gene numbers are the same as those in Table 4.

## Data Availability

The data presented in this study are available on request from the corresponding author. The draft genome sequence data are available at NCBI under GenBank accession number JAJNCP000000000.1.

## Supplementary Materials

The following supporting information can be downloaded at https://www.mdpi.com/article/10.3390/genes16070739/s1, Table S1: Physiological and biochemical features of *R. rhodochrous* IEGM 107; Table S2: Strains in the dataset and their type-based species and subspecies clustering.

## Abbreviations

The following abbreviations are used in this manuscript:

CDS	Coding sequence
CYP450	Cytochrome P450
*R*.	*Rhodococcus*
