# Supplementary material for: Genome Annotation and Catalytic Profile of Rhodococcus rhodochrous IEGM 107, Mono- and Diterpenoid Biotransformer
Source: Genes (Basel). 2025 Jun 26;16(7):739. doi: 10.3390/genes16070739 (PMC12294881; doi:10.3390/genes16070739)
Supplement: Supplementary file 1 [file genes-16-00739-s001.zip › Supplementary Materials Table S1.pdf]

Table S1. Physiological and biochemical features of *R. rhodochrous* IEGM 107

Feature	Manifestation (yes/no)
Synthetic mineral media support growth in the absence of vitamins (growth factors) and organic nitrogen compounds, provided that other sources of carbon, nitrogen and energy are provided	Yes
Synthetic mineral media support growth in the absence of organic nitrogen, provided other sources of carbon, nitrogen, energy and vitamin compounds are provided	Yes
Ammonium salts can be used as the sole source of nitrogen	Yes
Nitrates can serve as the only source of nitrogen	Yes
D-Glucose is metabolized	Yes
Lactate is metabolized	Yes
Lactate is metabolized under aerobic conditions	Yes
Nitrate is reduced to nitrite	No
Molecular nitrogen is used	Yes
Molecular oxygen is used	Yes
Hydrogen peroxide decomposes	Yes
Lactose is metabolized	No
Pyruvate is metabolized	Yes
Pyruvate is metabolized under aerobic conditions	Yes
D-Fructose is metabolized	Yes
D-Fructose is metabolized under aerobic conditions	Yes
L-Arabinose is used	No
D-Xylose is used	No
L-Rhamnose is used	No
D-Fructose is used	Yes
D-Mannose is used	Yes
L-Sorbose is used	No
Alpha-Methyl-D-Glucoside is used	No
Cellobiose is used	No
Lactose is used	No
Maltose is used	No
Sucrose is used	No
The acid is formed from D-glucose	Yes
The acid is formed from D-mannose	Yes
The acid is formed from sucrose	No
D-Fructose can be used as a sole carbon source	Yes
D-Glucose can be used as a sole carbon source	Yes
D-Mannose can be used as a sole carbon source	Yes
Sucrose can be used as a sole carbon source	No
Dulcite is used	No
D-Mannitol is used	Yes
D-Sorbitol is used	Yes
D-Mannitol can be used as a sole carbon source	Yes

D-Sorbitol can be used as a sole carbon source	Yes
Acetic acid is used	Yes
Caproic acid (hexanoic acid) is used	Yes
Formic acid is used	No
Palmitic acid is used	No
Succinic acid is used	Yes
Fumaric acid is used	Yes
DL-Lactic acid is used	Yes
Citric acid is used	Yes
Pyruvic acid is used	Yes
Alpha-Ketoglutaric acid is used	Yes
Phthalic acid is used	No
Isophthalic acid is used	No
Terephthalic acid is used	No
Benzoic acid is used	Yes
Metaoxybenzoic acid is used	No
Paraoxybenzoic acid is used	No
Phenylacetic acid is used	No
Acetic acid can be used as the sole carbon source	Yes
Caproic acid can be used as a sole carbon source	Yes
Propionic acid can be used as a sole carbon source	Yes
Succinic acid can be used as a sole carbon source	Yes
Fumaric acid can be used as a sole carbon source	Yes
DL-Lactic acid can be used as a sole carbon source	Yes
Citric acid can be used as the sole carbon source	Yes
Pyruvic acid can be used as a sole carbon source	Yes
Benzoic acid can be used as a sole carbon source	Yes
Gamma-Aminobutyric acid is used	No
Anthranilic acid is used	No
n-Butane is used as the sole carbon source	Yes
n-Decane is used as the sole carbon source	Yes
n-Dodecane is used as the sole carbon source	Yes
n-Hexadecane is used as the sole carbon source	Yes
