# Supplementary material for: Genome Annotation and Catalytic Profile of Rhodococcus rhodochrous IEGM 107, Mono- and Diterpenoid Biotransformer
Source: Genes (Basel). 2025 Jun 26;16(7):739. doi: 10.3390/genes16070739 (PMC12294881; doi:10.3390/genes16070739)
Supplement: Supplementary file 1 [file genes-16-00739-s001.zip › Supplementary Materials Table S2.pdf]

Table S2. Strains in the dataset and their type-based species and subspecies clustering

TYGS ID	Kind	Species cluster	Subspecies cluster	Preferred name	Deposit	Authority	Other deposits	Synonymous taxon names	Base pairs	Percent G+C	No. proteins	Goldstamp	Bioproject accession	Biosample accession	Assembly accession
13769	type strain	1	0	*Rhodococcus zopfii*	NBRC 100606	Stoecker et al. 1994 emend. Nouioui et al. 2018	CIP 104275; NRRL B-16942; ATCC 51349; DSM 44108; JCM 9919; T1	*Rhodococcus zopfii*	6 297 215	68,24	5885	Gp0023600	PRJDB309	SAMD00046765	GCA_001895025
13829	type strain	2	1	*Rhodococcus coprophilus*	NBRC 100603	Rowbotham and Cross 1979 emend. Nouioui et al. 2018	CIP 104178; NRRL B-16537; NCIMB 11211; ATCC 29080; DSM 43347; JCM 3200; NBRC 100603; NCTC 10994; IEGM 600; VKM Ac-571; CUB 687; LMG 5357; NCIB 11211	*Rhodococcus coprophilus*	4 549 097	66,88	4190	Gp0023589	PRJDB290	SAMD00046763	GCA_001895045
157919	type	2	1	*Rhodococcus*	NCTC	Rowbotha	CIP 104178;	*Rhodococcus*	4	66,83	4164		PRJEB6403	SAMEA44126	GCA_9004781

	strain			*coprophilus*	10994	m and Cross 1979 emend. Nouioui et al. 2018	NRRL B-16537; NCIMB 11211; ATCC 29080; DSM 43347; JCM 3200; NBRC 100603; NCTC 10994; IEGM 600; VKM Ac-571; CUB 687; LMG 5357; NCIB 11211	*coprophilus*	579834					84	15
157921	type strain	3	7	*Rhodococcus rhodochrous*	NCTC10210	(Zopf 1891) Tsukamura 1974 emend. Nouioui et al. 2018	CIP 104376; NRRL B-16536; NRRL B-2149; ATCC 13808; CCUG 47165; DSM 43241; JCM 3202; IFO 16069; NBRC 16069; NCTC	*Rhodococcus rhodochrous; Staphylococcus rhodochrous*	5274756	68,17	4815		PRJEB6403	SAMEA4535765	GCA_900187265

							10210; IEGM 62; VKM Ac-1227; HAMBI 1959; LMG 5365								
158052	type strain	3	7	*Rhodococcus rhodochrous*	DSM 43241	(Zopf 1891) Tsukamura 1974 emend. Nouioui et al. 2018	CIP 104376; NRRL B-16536; NRRL B-2149; ATCC 13808; CCUG 47165; DSM 43241; JCM 3202; IFO 16069; NBRC 16069; NCTC 10210; IEGM 62; VKM Ac-1227; HAMBI 1959; LMG 5365	*Rhodococcus rhodochrous; Staphylococcus rhodochrous*	5 177 761	68,24	4781		PRJNA306614	SAMN04357317	GCA_001646825
4056	type strain	3	7	*Rhodococcus rhodochrous*	NBRC 16069	(Zopf 1891) Tsukamura 1974 emend.	CIP 104376; NRRL B-16536; NRRL B-2149;	*Rhodococcus rhodochrous; Staphylococcus rhodochrous*	5 201 718	68,24	4784	Gp0023812	PRJDB303	SAMD00034188	GCA_001047055

						Nouioui et al. 2018	ATCC 13808; CCUG 47165; DSM 43241; JCM 3202; IFO 16069; NBRC 16069; NCTC 10210; IEGM 62; VKM Ac-1227; HAMBI 1959; LMG 5365								
U985682	user strain	3	7	JAJNCP01.1 a nt					5 730 104	67,81	5338				
11447	type strain	4	2	*Rhodococcus biphenylivorans*	TG9	Su et al. 2015	CGMCC 1.12975; KCTC 29673; MCCC 1K00286	*Rhodococcus biphenylivorans*	5 034 221	68,05	4395	Gp0359799	PRJNA390866	SAMN07251647	GCA_003288095
2548	type strain	4	2	*Rhodococcus pyridinivorans*	DSM 44555	Yoon et al. 2000 emend. Nouioui et al. 2018	KCTC 0647BP; JCM 10940; NBRC 100608; KCCM 80005; PDB9	*Rhodococcus pyridinivorans*	5 262 484	67,85	4848	Gp0117007	PRJNA303862	SAMN04490240	GCA_900105195

25580	type strain	5	3	*Rhodococcus sacchari*	Z13	Zang et al. 2025	JCM 35797; CCTCC AB 2022327	*Rhodococcus sacchari*	4 532 339	69,81	4095		PRJNA224116	SAMN31157772	GCF_025837095
5382	type strain	6	4	*Rhodococcus gordoniae*	DSM 44689	Jones et al. 2004 emend. Nouioui et al. 2018	JCM 12658; NCTC 13296; W 4937	*Rhodococcus gordoniae*	4 824 352	67,95	4419	Gp0150350	PRJNA224116	SAMN04357310	GCF_001646655
5383	type strain	7	5	*Rhodococcus phenolicus*	DSM 44812	Rehfuss and Urban 2006 emend. Nouioui et al. 2018	NRRL B-24323; JCM 14914; G2P	*Rhodococcus phenolicus*	6 283 557	68,36	5932	Gp0150351	PRJNA224116	SAMN04357313	GCF_001646785
753	type strain	8	6	*Rhodococcus chondri*	CC-R104	Girão et al. 2024	UCCCB 171; LMG 33233	*Rhodococcus chondri*	5 332 803	67,02	5093		PRJNA999295	SAMN36781230	GCA_036327435
